# The impact of nursing interns’ intolerance of uncertainty on professional identity: the mediating role of career resilience

**DOI:** 10.3389/fmed.2026.1820199

**Published:** 2026-06-08

**Authors:** Yaqi Wu, Hejia Wan, Lisha Zhao, Jie Liu, Lulu Zhang, Xin Wang, Jing Mao, Xiaolei Jing

**Affiliations:** 1 School of Nursing (Nursing School of Smart Healthcare Industry), Henan University of Chinese Medicine, Zhengzhou, Henan, China; 2 Department of Nursing, The First Affiliated Hospital of Henan University of Chinese Medicine, Zhengzhou, Henan, China

**Keywords:** career resilience, intolerance of uncertainty, mediation effect analysis, nursing interns, professional identity

## Abstract

**Background:**

With the rapid development of the nursing industry, the stability and professional competence of nursing personnel have become increasingly important. Professional identity, as a key factor influencing the development of nursing personnel, is crucial to the construction and development of the nursing workforce. Especially during the clinical internship phase, nursing interns face many sources of uncertainty, which may negatively affect their professional identity. However, some interns are able to maintain a high level of professional identity in uncertain environments, which may be closely related to their career resilience. This study aims to explore the mediating role of career resilience between intolerance of uncertainty and nursing interns’ professional identity, providing theoretical support and intervention strategies for enhancing interns’ professional identity.

**Methods:**

A convenience sampling method was used to select 298 nursing students who had completed 8 months of internship at a tertiary hospital in Zhengzhou from February to April 2025 as the study subjects. A general demographic questionnaire, the short version of the Intolerance of Uncertainty Scale (IUS-12), the Career Resilience Scale (CRS), and the Nursing Student Professional Identity Questionnaire (PIQN) were used for the survey. Descriptive statistics of all variables were conducted using SPSS 27.0 software to understand the basic characteristics of the sample. Pearson correlation analysis was used to test the correlations between intolerance of uncertainty (IU), career resilience (CRS), and professional identity (PIQN). The Process macro model 4 (developed by Hayes) was used for mediation effect analysis to test the mediating role of career resilience (CRS) between intolerance of uncertainty (IU) and professional identity (PIQN).

**Results:**

The total score for intolerance of uncertainty in nursing interns was (38.57 ± 7.20), for career resilience (19.98 ± 2.71), and for professional identity (61.04 ± 9.30). Intolerance of uncertainty was negatively correlated with professional identity(*r* = −0.26, *p* < 0.001) and with career resilience (*r* = −0.31, *p* < 0.001), while career resilience was positively correlated with professional identity (*r* = 0.48, *p* < 0.001). Mediation analysis revealed that career resilience partially mediated the relationship between intolerance of uncertainty and professional identity [indirect effect = −0.17, 95% CI (−0.26, −0.10)], accounting for 52.64% of the total effect.

**Conclusion:**

Intolerance of uncertainty directly negatively affects nursing interns’ professional identity, while career resilience plays a partial mediating role in buffering the negative impact of intolerance of uncertainty on professional identity. This suggests that nursing educators should specifically help nursing interns actively cope with uncertain situations and strengthen the development of career resilience, thereby enhancing their professional identity.

## Introduction

Professional identity (PI) refers to an individual’s self-perception and evaluation of their career goals, competencies, personal interests, and values (1). In the field of nursing, the professional identity of nursing students affects their transition from student nurses to professional nurses and their willingness to remain in the nursing profession (2). High professional identity has a positive impact on the academic success, learning motivation and professional development of nursing students, and it is also the most important factor influencing their future career choices and professional sense of achievement (3). Conversely, nursing students with a lower sense of professional identity may experience uncertainty and confusion about their career, leading to a lack of motivation in their studies and even choosing to leave the nursing profession after graduation (4). Professional identity not only affects the career development of individual nursing students, but also has a significant impact on the stability and development of the entire nursing industry (4). Therefore, in nursing education, the cultivation of professional identity is particularly important (5). However, during the internship stage, this period is a crucial transitional period for nursing students from theory to practice (6). When facing real clinical situations, intern nursing students may experience a high degree of uncertainty, such as changes in patients’ conditions, the uncertainty of clinical decisions, and the differences between clinical practice and academia. These uncertainties may challenge the professional identity of nursing students and lead to a decline in their interest and commitment to the profession (7). Therefore, nursing educators should focus on helping nursing students develop a positive sense of professional identity during the internship stage (8).

**Figure 1:**
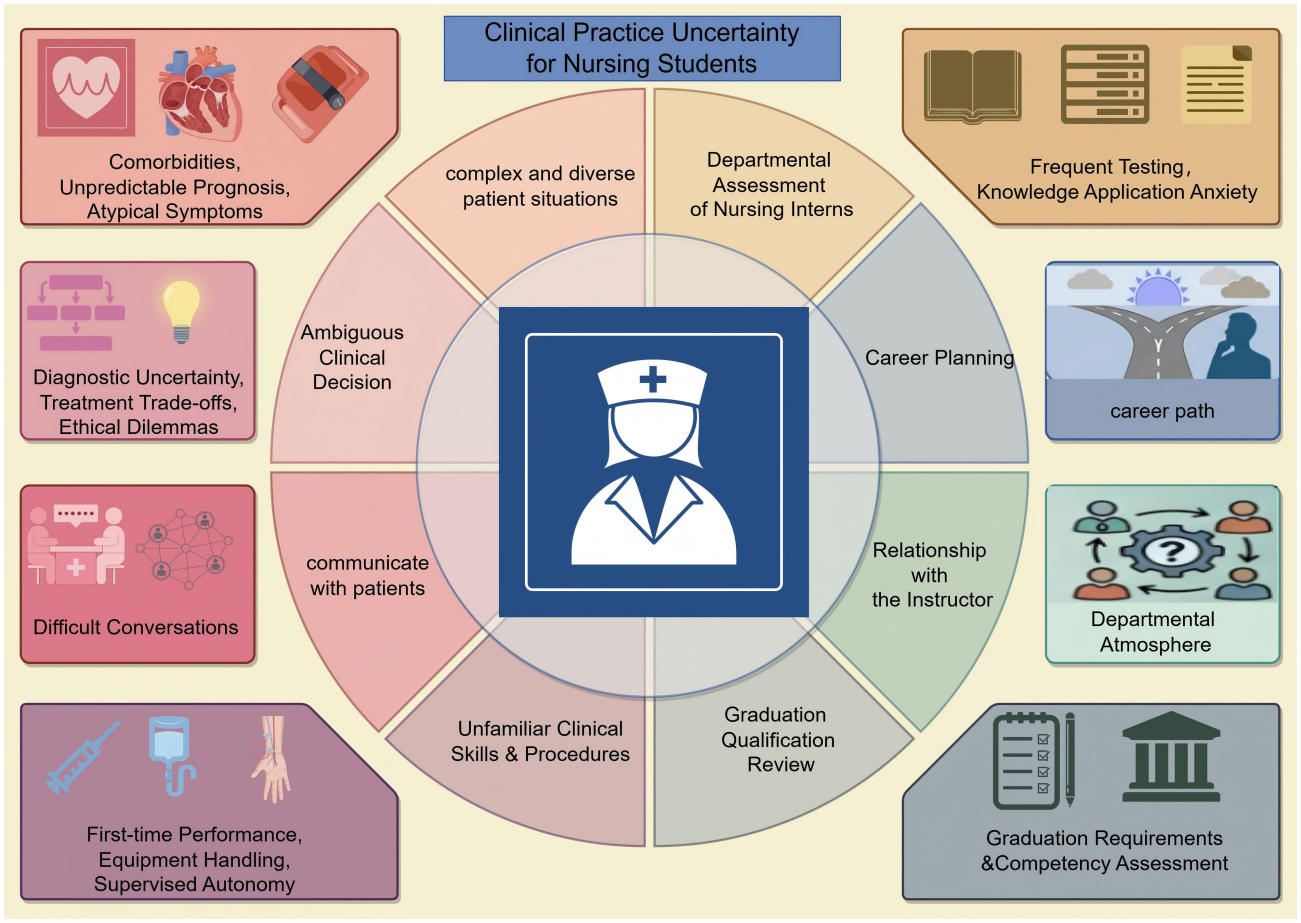
Sources of clinical practice uncertainty for nursing students.

At present, undergraduate medical courses increasingly emphasize cultivating graduates’ ability to cope with uncertainties. This stems from the fact that uncertainties are ubiquitous and have far-reaching impacts in clinical practice (9). Intern nursing students often encounter complex and unpredictable situations in clinical work, such as rapid changes in patients’ conditions, the selection of treatment plans, and the uncertainty of clinical decisions (10). As illustrated in Figure 1, the clinical practice uncertainty for nursing students is multidimensional, encompassing ambiguous clinical decisions, unfamiliar clinical skills, departmental assessments, and future career planning. Research shows that uncertainty in the clinical environment is one of the most common challenges faced by intern nursing students (11). In addition, many nursing students feel confused about the uncertainty of their future career development, especially their concerns about the salary, promotion and career prospects of the nursing profession, which have further shaken their professional identity (12). Intolerance of uncertainty (IU) is a cognitive bias that perceives, interprets and reacts to uncertain situations or events, which affects individuals’ cognition, emotion and behavioral responses (13). Specifically, individuals with a higher IU tend to view uncertainty as a negative and threatening factor (13). Research has found that intolerance of uncertainty (IU) is closely related to emotional distress, anxiety, and cognitive dissonance, often leading to strong anxiety and avoidance responses in ambiguous situations, which in turn affect decision-making and coping strategies (14). Particularly during the internship phase, nursing students, due to a lack of sufficient clinical experience, have a lower tolerance for uncertainty and are prone to emotional fluctuations when faced with complex clinical situations, which may negatively impact their professional identity (15).

However, although existing studies have explored the impact of intolerance of uncertainty on nursing students’ mental health (16), few have focused on how it affects their professional identity. More importantly, current research predominantly focuses on the impact of a single variable, neglecting the potential moderating role of career resilience. Career resilience (CR) refers to an individual’s ability to recover to a normal work state through proactive coping and adjustment when faced with difficulties and setbacks in their career (17). Career resilience is not only an important psychological resource, but also considered a protective factor for individuals in coping with career stress, uncertainty, and overcoming professional challenges (17). Existing research has shown that career resilience can help individuals enhance their ability to cope with challenges, improving job satisfaction and career adaptability (18). However, current research on the role of career resilience in nursing interns is relatively scarce.

The Conservation of Resources Theory (COR) provides theoretical support for understanding the role of career resilience in this process. This theory posits that individuals cope with stressors effectively by mobilizing and accumulating resources (such as emotional support and psychological resilience), thereby achieving psychological and workplace adaptation (19). For nursing interns, career resilience, as an important psychological resource, can help them maintain a positive professional identity and alleviate anxiety and stress caused by uncertainty in clinical practice. However, research on the mechanisms of career resilience in nursing students’ professional identity is still insufficient, particularly regarding how career resilience moderates the relationship between intolerance of uncertainty and professional identity, a field that still lacks systematic and in-depth exploration.

Therefore, this study aims to explore the mediating role of career resilience between intolerance of uncertainty and professional identity. By revealing how career resilience moderates the effect of intolerance of uncertainty on professional identity, this study not only fills a research gap in the field of nursing but also provides a theoretical basis and intervention directions for nursing education. Specifically, this study will offer references for nursing educators in fostering nursing students’ career resilience and enhancing their ability to cope with uncertain situations, while providing practical psychological support strategies for nursing interns to strengthen their professional identity and career development stability.

## Methods

### Participants

According to the sample size calculation formula N = (Z_*α*/2_*σ*/*δ*)^2^ (20), the pre-survey results of 30 nursing interns showed a professional identity score of (59.30 ± 10.21). Therefore, with *α* = 0.05, *σ* = 10.21, and *δ* = 1.5, the sample size required at least 178 subjects. Considering 10–20% invalid questionnaires, the final sample size was calculated to be at least 195 subjects. Additionally, given the need for the construction of a mediation model, the sample size should be at least 200 (21). In this study, a total of 298 nursing interns were included. A convenience sampling method was used to select nursing interns from a tertiary hospital in Zhengzhou as the study subjects. Inclusion criteria: nursing interns currently on duty; internship duration longer than 8 months; informed consent and voluntary participation in the study. Exclusion criteria: individuals unable to complete the questionnaire due to personal reasons or other circumstances.

### Study design and procedure

This study was approved by the Ethics Committee of the First Affiliated Hospital of Henan University of Traditional Chinese Medicine and was conducted through on-site surveys. From February to April 2025, the survey was distributed in the departments using electronic questionnaires by members of the research team. Informed consent was obtained from the participants before the survey, and during the survey, the investigator provided timely explanations for any unclear content. The electronic questionnaire included a standardized instruction explaining the purpose, usage, completion method, and precautions for the survey. All options were set as mandatory questions, and each IP address was allowed to submit only one response. A total of 310 questionnaires were collected, and those with excessively high consistency were excluded. Of these, 298 valid questionnaires were retained, resulting in an effective response rate of 96.1%.

### Measures

#### General information survey questionnaire

A questionnaire designed by the researchers was used for the survey, including information on the participants’ place of origin, education level, gender, and reasons for choosing the nursing profession.

#### Intolerance of uncertainty

The short version of the Intolerance of Uncertainty Scale (IUS-12), developed by Canadian scholars Carleton et al. (22), was translated and revised into Chinese by Zhang et al. (23) to assess intolerance of uncertainty among Chinese university students. The scale consists of 12 items, including two dimensions: anticipatory anxiety and inhibitory anxiety. A Likert 5-point scale is used, ranging from “strongly disagree” to “strongly agree,” scored 1 to 5. A higher total score indicates a higher level of intolerance of uncertainty. The internal consistency coefficient of the revised Chinese version of the IUS-12 ranged from 0.704 to 0.878. In this study, the Cronbach’s *α* coefficient of the scale was 0.847.

#### Nursing students’ professional identity

The Professional Identity Questionnaire for Nursing Students (PIQN) was developed by Hao (24) to measure nursing students’ professional identity. The questionnaire includes 5 dimensions: professional self-concept (6 items), social comparison and self-reflection (3 items), job retention and turnover benefits (4 items: 5, 8, 10, 14), career choice autonomy (2 items: 4, 12), and social persuasion (2 items: 2, 3). The scale uses a Likert 5-point scoring system, ranging from “strongly disagree” to “strongly agree,” with scores from 1 to 5, where item 12 is reverse scored. The total score of the questionnaire is 85, with higher scores indicating a higher level of professional identity. The Cronbach’s *α* coefficient is 0.827. In this study, the Cronbach’s α coefficient of the scale was 0.905.

#### Career resilience

The Career Resilience Scale (CRS) was developed by American scholar London (25) and adapted into Chinese by Wang Qiaoling, used to assess an individual’s career resilience. The scale consists of 5 items and is unidimensional, using a Likert 5-point scale from “strongly disagree” to “strongly agree,” scored from 1 to 5, with a total score ranging from 5 to 25. Higher scores indicate higher levels of career resilience. The Cronbach’s α coefficient of the scale is 0.933, and in this study, the Cronbach’s α coefficient of the scale was 0.793.

### Data analysis

Data were analyzed using SPSS 27.0 software. For normally distributed continuous variables, the mean ± standard deviation was used for description. Categorical data were presented using frequency and percentage. Harman’s single-factor test was used to check for common method bias. Pearson correlation analysis was used to assess the correlations between intolerance of uncertainty, career resilience, and professional identity. In the analyses, intolerance of uncertainty was operationalized as the total score of the IUS-12, a 12-item scale with scores ranging from 12 to 60, where higher scores reflect greater intolerance of uncertainty. Professional identity was operationalized as the total score of the PIQN, a 17-item scale with scores ranging from 17 to 85, where higher scores reflect stronger professional identity. Career resilience was operationalized as the total score of the CRS, a unidimensional 5-item scale with scores ranging from 5 to 25, where higher scores reflect greater career resilience. Mediating effects were analyzed using Model 4 from the Process 4.1 version of the SPSS macro program developed by Hayes. The Bootstrap method (with 5,000 resamples of the original data) was used to test the mediating effect, with a significance level set at *α* = 0.05.

## Results

The nursing students were grouped according to their place of origin, education level, gender, and reasons for choosing the nursing profession. Skewness and kurtosis values for all continuous variables were within acceptable ranges, indicating approximate normality. Specifically, the values were as follows: intolerance of uncertainty (skewness = 0.205, kurtosis = −0.070), career resilience (skewness = 0.130, kurtosis = −0.402), and professional identity (skewness = 0.047, kurtosis = 0.034). These results support the assumption of normality, justifying the use of parametric statistical analyses. The results of the one-way analysis showed that nursing students with different reasons for choosing the nursing profession had statistically significant differences in their professional identity scores (*p* < 0.05), as shown in Table 1.

**Table 1:** Comparison of professional identity scores in nursing students with different characteristics (*N* = 298).

Variable	Number (%)	Professional identity score (mean ± SD)	*t*/*F*	*p*
Place of origin			1.550	0.214
Rural	155 (52.0)	60.40 ± 8.85		
Urban	143 (48.0)	61.74 ± 9.75		
Education level			2.036	0.155
Associate degree	185 (62.1)	61.64 ± 9.40		
Bachelor’s degree	113 (37.9)	60.06 ± 9.09		
Gender			0.138	0.711
Male	57 (19.1)	60.63 ± 10.55		
Female	241 (80.9)	61.14 ± 9.00		
Reasons for choosing nursing			17.027	<0.001
Career prospects	135 (45.3)	62.23 ± 7.91		
Personal interest	50 (16.8)	67.82 ± 9.09		
Family factors	53 (17.8)	57.79 ± 8.68		
Major adjustment	27 (9.1)	54.96 ± 7.74		
Other	33 (11.1)	56.12 ± 9.57		

Harman’s single-factor test was used to perform unrotated exploratory factor analysis on all items of intolerance of uncertainty, career resilience, and professional identity in nursing students. The results showed that there were 7 common factors with eigenvalues greater than 1, and the first factor explained 27.95% of the variance, which is below the 40% critical threshold (26). This indicates that there is no significant common method bias in this study.

The total scores and mean scores for each item of intolerance of uncertainty, career resilience, and professional identity in nursing students are shown in Table 2.

**Table 2:** Scores of intolerance of uncertainty, career resilience, and professional identity in nursing students (*N* = 298, Mean ± SD).

Category	Number of items	Mean score (x ± s)	Total score
Intolerance of uncertainty	12	3.21 ± 0.60	38.57 ± 7.20
Expectancy dimension	7	3.27 ± 0.62	22.89 ± 4.31
Inhibition dimension	5	3.13 ± 0.65	15.69 ± 3.27
Career resilience	5	4.00 ± 0.54	19.98 ± 2.71
Professional identity	17	3.59 ± 0.55	61.04 ± 9.30
Professional self-concept	6	3.56 ± 0.69	21.34 ± 4.15
Social comparison & self-reflection	3	3.89 ± 0.56	11.68 ± 1.68
Job retention vs. leaving benefits	4	3.33 ± 0.68	13.34 ± 2.71
Career choice autonomy	2	3.45 ± 0.82	6.90 ± 1.65
Social persuasion	2	3.90 ± 0.79	7.79 ± 1.58

The results of the Pearson correlation analysis showed that intolerance of uncertainty in nursing students was negatively correlated with professional identity (*r* = −0.255, *p* < 0.001), negatively correlated with career resilience (*r* = −0.309, *p* < 0.001), and career resilience was positively correlated with professional identity (*r* = 0.482, *p* < 0.001).

In this study, intolerance of uncertainty was entered as the independent variable, career resilience as the mediator, and professional identity as the dependent variable. The variable “reasons for choosing the nursing profession,” which demonstrated statistical significance in the preliminary univariate analysis, was included as a covariate. Hayes’ PROCESS macro (Model 4) was utilized to test the mediating effect of career resilience between intolerance of uncertainty and professional identity.

The linear regression analyses embedded within the mediation framework revealed that intolerance of uncertainty significantly and negatively predicted professional identity (*β* = −0.25, *p* < 0.001), explaining 15% of the variance (*R*^2^ = 0.15). Furthermore, intolerance of uncertainty significantly and negatively predicted the mediator, career resilience (*β* = −0.31, *p* < 0.001). After simultaneously entering career resilience into the model, both intolerance of uncertainty (*β* = −0.12, *p* = 0.018) and career resilience (*β* = 0.43, *p* < 0.001) remained significant predictors of professional identity, with the combined model explaining 32% of the variance (*R*^2^ = 0.32). These results provided the foundation for the subsequent mediation analysis.

The unstandardized regression coefficient for the effect of intolerance of uncertainty on professional identity decreased in magnitude from −0.33(total effect) to −0.16 (direct effect) after including career resilience in the model. These results indicate that career resilience plays a partial mediating role between intolerance of uncertainty and professional identity, as shown in Table 3.

**Table 3:** Mediation effect test.

Predictors	Model 1		Model 2		Model 3	
	*β*	*t*	*β*	*t*	*β*	*t*
Reason for choosing nursing	−0.30	−5.54***	−0.05	−0.83	−0.28	−5.76***
Intolerance of uncertainty	−0.25	−4.73***	−0.31	−5.58***	−0.12	−2.37*
Career resilience					0.43	8.54***
*R* ^2^	0.15		0.10		0.32	
*F*	26.69***		15.96***		46.43***	

Model 1: Intolerance of Uncertainty Predicts Professional Identity; Model 2: Intolerance of Uncertainty Predicts Career Resilience; Model 3: Intolerance of Uncertainty and Career Resilience Predict Professional Identity. *p < 0.05, **p < 0.01, ***p < 0.001.

Further bias-corrected Bootstrap analysis revealed that the indirect (mediation) effect was −0.17 [95% CI (−0.26, −0.10)]. Since the 95% confidence interval does not include zero, the mediating role of career resilience is statistically significant. In summary, the total effect was −0.33, the direct effect was −0.16 [95% CI (−0.28, −0.03)], and the mediation effect accounted for 52.64% of the total effect.

This indicates that career resilience plays a significant mediating role in the effect of intolerance of uncertainty on professional identity, as shown in Table 4.

**Table 4:** Total effect, direct effect, and mediation effect decomposition.

Effect type	Effect value	Boot SE	LLCI	ULCI	Effect Proportion
Total effect	−0.33	0.07	−0.46	−0.19	
Direct effect	−0.16	0.07	−0.28	−0.03	47.36%
Mediation effect	−0.17	0.04	−0.26	−0.10	52.64%

## Discussion

This study explores the role of intolerance of uncertainty (IU) and career resilience in the formation of professional identity among nursing interns. The results show that the mean score for intolerance of uncertainty among nursing interns was 38.57 ± 7.20, which is moderately high. This is similar to the findings of Yesilot et al. (16) (38.84 ± 10.01) in a study on nursing students. The may be due to the different internship stages of the study populations. Our study participants were all in the later stages of their internship, during which nursing students not only face uncertainty in clinical practice but also confront multiple stressors such as licensing exams, graduation qualification reviews, and job competition (27). These stressors increase the perception of uncertainty about the future, causing heightened anxiety and stress, which in turn affects their tolerance for uncertainty. Therefore, the multiple uncertain situations in the later stages of internships may make nursing students more psychologically sensitive and increase their anxiety about uncertainty. Based on this, nursing managers should pay attention to the multiple uncertainties faced by interns in the later stages of their internship, actively communicate with them, and provide professional guidance. Additionally, cognitive-behavioral interventions should be considered to help nursing students correctly perceive and accept uncertainty. Furthermore, the school should improve the employment guidance and service system (28) to better support nursing students in coping with career uncertainties.

The results of this study show that the total career resilience score for nursing interns was 19.98 ± 2.71, with an average item score of 4.00 ± 0.54. This finding indicates that despite facing a complex and challenging clinical environment, most nursing interns possess a strong capacity to adapt, bounce back from adversity, and maintain a positive attitude toward their profession. This result aligns with the findings of Mayor-Silva et al. (29), who also reported moderate-to-high resilience levels among nursing students during their clinical practicum. Several factors might explain this positive outcome. First, nursing interns in this study are likely undergoing intensive clinical preceptorship. Supportive mentoring from experienced clinical instructors can significantly buffer workplace stress and foster interns’ psychological resilience. Second, as newcomers to the clinical setting, interns may still possess high levels of enthusiasm and professional idealism, which serve as internal protective resources when encountering initial setbacks. Furthermore, the systematic psychological education provided by nursing schools in recent years may have equipped these students with effective coping strategies before entering clinical practice. Given its crucial role as a buffer against clinical uncertainties—as demonstrated in our mediation analysis—maintaining and further cultivating this high level of career resilience is essential for facilitating interns’ successful transition into registered nurses.

This study also shows that the total professional identity score for nursing interns was 61.04 ± 9.30, which is moderately high, higher than the result found by Leng et al. (30) (57.29 ± 11.51). This difference may be due to the fact that the participants in this study were all in the later stages of their internship. At this stage, nursing interns have generally completed the initial transition from students to quasi-professionals (31) and gradually accumulate experience in clinical practice, allowing them to make more independent judgments and decisions (32), thereby enhancing their professional identity. However, within the dimensions of professional identity, the scores for career choice autonomy (3.45 ± 0.82) and perceived benefits of staying or leaving the profession (3.33 ± 0.68) were relatively low. This suggests that nursing interns may have a passive attitude toward choosing the nursing profession and may lack a deep understanding of the long-term value of the nursing profession. They may not fully recognize the profound significance of nursing in terms of personal growth, professional development, and social contribution. This shortsightedness in professional identity may stem from insufficient promotion of the value of the profession in nursing education and a lack of career guidance in the internship setting. In particular, during the internship phase, interns’ professional identity is often limited by their current clinical experiences and they may not fully realize the long-term significance of the nursing profession. At the same time, the low scores for perceived benefits of staying or leaving the profession indicate that, although nursing students recognize that leaving the nursing field might result in emotional loss, this awareness is primarily driven by the cost of learning investment, rather than emotional attachment to the profession itself. This suggests that nursing managers should strengthen the development of nursing students’ career choice autonomy and reinforce the promotion of the profession’s value. By offering lectures on industry development trends, career development planning guidance (33) and creating supportive internship environments, nursing students can be encouraged to explore their career paths autonomously, thereby enhancing their professional belonging and sense of responsibility.

The results of this study revealed a statistically significant negative correlation between intolerance of uncertainty and professional identity, although the magnitude of this correlation was relatively weak. This finding is not unexpected, as professional identity is a complex, multidimensional construct shaped by a broad range of personal, educational, and contextual factors, of which intolerance of uncertainty is only one. Importantly, the mediation analysis demonstrated that 52.64% of the total effect of intolerance of uncertainty on professional identity operated indirectly through career resilience, suggesting that the relationship between these two variables is more meaningfully captured through the mediating pathway than through the direct association alone. Intolerance of uncertainty is a stable psychological tendency in which individuals tend to interpret ambiguous or uncertain information as a threat, leading to anxiety, avoidance, and hesitation (13). Nursing students with higher intolerance of uncertainty tend to experience anxiety, confusion, or a sense of helplessness when faced with uncertainty in clinical or future employment situations. These emotional and psychological states may make them feel disturbed by the uncertainty of their career development, thus reducing their professional identity. Furthermore, intolerance of uncertainty was significantly and negatively correlated with career resilience (*r* = −0.31, *p* < 0.001). Career resilience reflects an individual’s ability to adapt and recover under stress and challenges (36). According to the stress-coping theory, nursing students with higher intolerance of uncertainty are more prone to negative cognitive and emotional responses; consequently, they may struggle to mobilize internal resources for effective psychological adjustment, which compromises their adaptability in uncertain situations. Moreover, career resilience was positively correlated with professional identity, reflecting a moderate-to-strong association according to Cohen’s (1988) benchmarks. This finding suggests that career resilience plays a meaningful and substantial role in shaping professional identity among nursing interns. This is further supported by our mediation analysis, which demonstrated that career resilience accounted for 52.64% of the total effect of intolerance of uncertainty on professional identity(*r* = 0.48, *p* < 0.001), which is consistent with the findings of Babamohamadi et al. (37) and Aryuwat et al. (34),who highlighted the positive link between professional identity and psychological resilience, noting that professional identity can be enhanced through the development of career resilience (35). As a vital psychological resource, career resilience plays a key role in stressful environments. Specifically, nursing students with higher career resilience possess stronger emotional regulation abilities, enabling them to effectively manage negative emotions and maintain a positive mindset when facing clinical challenges and uncertainties. These students are also more proactive in seeking support and solving problems, which enhances their professional competence and self-efficacy, ultimately solidifying their professional identity (38).

The results of this study demonstrate that career resilience partially mediates the relationship between intolerance of uncertainty and professional identity, with an indirect effect of −0.17 [95% CI (−0.26, −0.10)], accounting for 52.64% of the total effect. This indicates that intolerance of uncertainty not only has a direct negative predictive effect on professional identity but also influences professional identity indirectly through career resilience. According to the stress-coping model, when individuals face stressors in the external environment (such as clinical uncertainty, future employment pressure, etc.), they generate stress responses and adapt through various coping resources and strategies. However, when individuals lack tolerance for uncertainty, they tend to view uncertain or ambiguous information as a threat, leading to anxiety and avoidance, which negatively impacts their professional development and undermines their professional identity. The Conservation of Resources (COR) theory suggests that individuals, when facing stress, tend to use existing resources (such as psychological, emotional, and social support) to cope with stressors and seek new resources to maintain psychological and emotional balance. For nursing interns, multiple uncertainties in clinical practice (such as changes in patient condition, complex clinical decision-making, and uncertainty about future career development) create anxiety, avoidance, and other negative emotional states. The COR theory reveals how career resilience, as a critical psychological resource, acts as a buffer between intolerance of uncertainty and professional identity. When nursing students have high career resilience, despite facing high levels of uncertainty, they can still effectively mobilize psychological and emotional resources through strategies such as emotional regulation, positive coping, and meaning reconstruction, maintaining their professional identity and sense of belonging. Therefore, this study further confirms the negative impact of intolerance of uncertainty on professional identity and reveals the mediating role of career resilience between the two, providing theoretical support for enhancing nursing students’ professional identity. Based on this, nursing managers and educators should pay attention to nursing students’ intolerance of uncertainty, guide them using the stress-coping model and the COR theory, focus on cultivating career resilience, and provide systematic career planning and psychological support to help nursing students enhance their adaptability and psychological resilience when facing uncertainty. This will, in turn, improve their identification with and commitment to the nursing profession and promote the sustainable and healthy development of the nursing workforce.

## Conclusion

The results of this study show that intolerance of uncertainty not only has a direct effect on professional identity but also influences professional identity indirectly through career resilience. Nursing managers should pay attention to nursing interns with higher intolerance of uncertainty scores and address their psychological adjustment and career resilience training to enhance their ability to cope with uncertainty, thereby promoting the formation and consolidation of their professional identity.

### Limitations

This study employed a convenience sampling method and only selected nursing interns from a tertiary hospital in Zhengzhou as the research subjects. As a non-probability sampling approach, convenience sampling may introduce selection bias and limit the inferential generalizability of the findings to broader nursing intern populations. Future research should consider adopting probability-based sampling strategies across multiple institutions and regions to enhance representativeness. Additionally, the use of multiple data collection methods is recommended to facilitate study replication and validation. Furthermore, as a cross-sectional study, this research only collected data at a single time point and cannot capture changes in variables over time. Future studies could adopt longitudinal designs to track the dynamic relationships between intolerance of uncertainty, career resilience, and professional identity among nursing interns.

## Data Availability

The original contributions presented in the study are included in the article/supplementary material, further inquiries can be directed to the corresponding author.
